# Time-Wise Change in Neck Pain in Response to Rehabilitation with Specific Resistance Training: Implications for Exercise Prescription

**DOI:** 10.1371/journal.pone.0093867

**Published:** 2014-04-07

**Authors:** Mette K. Zebis, Christoffer H. Andersen, Emil Sundstrup, Mogens T. Pedersen, Gisela Sjøgaard, Lars L. Andersen

**Affiliations:** 1 Arthroscopic Center Amager, Copenhagen University Hospital, Amager, Copenhagen S, Denmark; 2 Gait Analysis Laboratory, Copenhagen University Hospital, Hvidovre, Hvidovre, Denmark; 3 National Research Centre for the Working Environment, Copenhagen Ø, Denmark; 4 Department of Nutrition, Exercise and Sports, University of Copenhagen, Copenhagen N, Denmark; 5 Institute of Sports Science and Clinical Biomechanics, University of Southern Denmark, Odense, Denmark; The University of Queensland, Australia

## Abstract

**Purpose:**

To determine the time-wise effect of specific resistance training on neck pain among industrial technicians with frequent neck pain symptoms.

**Methods:**

Secondary analysis of a parallel-group cluster randomized controlled trial of 20 weeks performed at two large industrial production units in Copenhagen, Denmark. Women with neck pain >30 mm VAS (N = 131) were included in the present analysis. The training group (N = 77) performed specific resistance training for the neck/shoulder muscles three times a week, and the control group (N = 54) received advice to stay active. Participants of both groups registered neck pain intensity (0–100 mm VAS) once a week.

**Results:**

Neck pain intensity was 55 mm (SD 23) at baseline. There was a significant *group by time* interaction for neck pain (F-value 2.61, P<0.001, DF = 19). Between-group differences in neck pain reached significance after 4 weeks (11 mm, 95% CI 2 to 20). The time-wise change in pain showed three phases; a rapid decrease in the training group compared with the control group during the initial 7 weeks, a slower decrease in pain during the following weeks (week 8–15), and a plateau during the last weeks (week 16–20). Adherence to training followed a two-phase pattern, i.e. weekly participation rate was between 70–86% during the initial 7 weeks, dropping towards 55–63% during the latter half of the training period.

**Conclusion:**

Four weeks of specific resistance training reduced neck pain significantly, but 15 weeks is required to achieve maximal pain reduction. The time-wise change in pain followed a three-phase pattern with a rapid effect during the initial 7 weeks followed by a slower but still positive effect, and finally a plateau from week 15 and onwards. Decreased participation rate may explain the decreased efficacy during the latter phase of the intervention.

## Introduction

Neck and shoulder pain is one of the most frequent health complaints among adults [1], [2]. In the general population, chronic neck pain is reported in up to 22% of women and 16% of men [3]. A Danish study among more than 5000 representative employees showed that approximately one third of adult workers suffer from moderate to severe neck pain [4], [5].The seriousness is emphasized by the fact that socioeconomic consequences of chronic disorders in the neck and shoulders in terms of disability, sick leave, and early retirement are considerable [4], [6], [7].

Different types of physical exercise have been evaluated as treatment of neck and shoulder pain [8]–[11], and there is strong evidence that strength training reduces neck pain [12]–[14]. However, fulfilling public recommendations of physical activity is challenging for the majority of people, often due to lack of time [15].

It has been shown that strength training implemented at the workplace is very effective in reducing neck pain [16], [17]. However, workplace interventions involving physical activity during working hours are typical very costly [18], and the effectiveness is evidently depending on adherence to exercise [19]. Thus, in respect to economical and personal barriers to exercise, one effective way to introduce physical activity to relieve neck pain at the workplace may be by prescribing an ‘exercise dose’, The perspective would be that an effective worksite ‘exercise dose’ could be implemented whenever needed in response to work related neck/shoulder pain.

Strength training exercises performed in accordance with the American College of Sports Medicine guidelines ensure a physiological training effect [20]. Time-wise physiological changes as neural adaptations and muscle hypertrophy typically are observed after only 6 and 12 weeks, respectively [21], [22]. In patients with chronic musculoskeletal pain, a change in pain intensity of 10 mm on a scale of 0 to 100 is considered the minimally important difference, and a change of 20 is considered to be moderately clinically meaningful [23]. However, few studies have evaluated the time-wise effectiveness of high-intensity strength training in reducing neck pain [16], [24]. In respect to prescribing an “exercise dose” it is essential to define the number of weeks necessary to achieve a clinical satisfying pain reduction.

This study investigates the time-wise change in neck pain during 20 weeks of specific strength training. We hypothesized that musculoskeletal pain in the neck will reach a plateau during 20 weeks of specific neck/shoulder strength training.

## Methods

### Study design

The present study is a secondary analysis of a parallel-group cluster randomized controlled trial of 20 weeks performed at two large industrial production units in Copenhagen, Denmark [25].

Exclusion criteria were pregnancy and serious health conditions such as previous trauma or injuries, life-threatening diseases and cardiovascular diseases.

### Ethics statement

We informed all participants about the purpose and content of the project and they gave written consent to participate in the study, which conformed to the Declaration of Helsinki and was approved by The Committees on Biomedical Research Ethics of the Capital Region of Denmark

(HC2008103). The study was registered in ClinicalTrials.gov (NCT01071980).

### Participants

**Figure 1:**
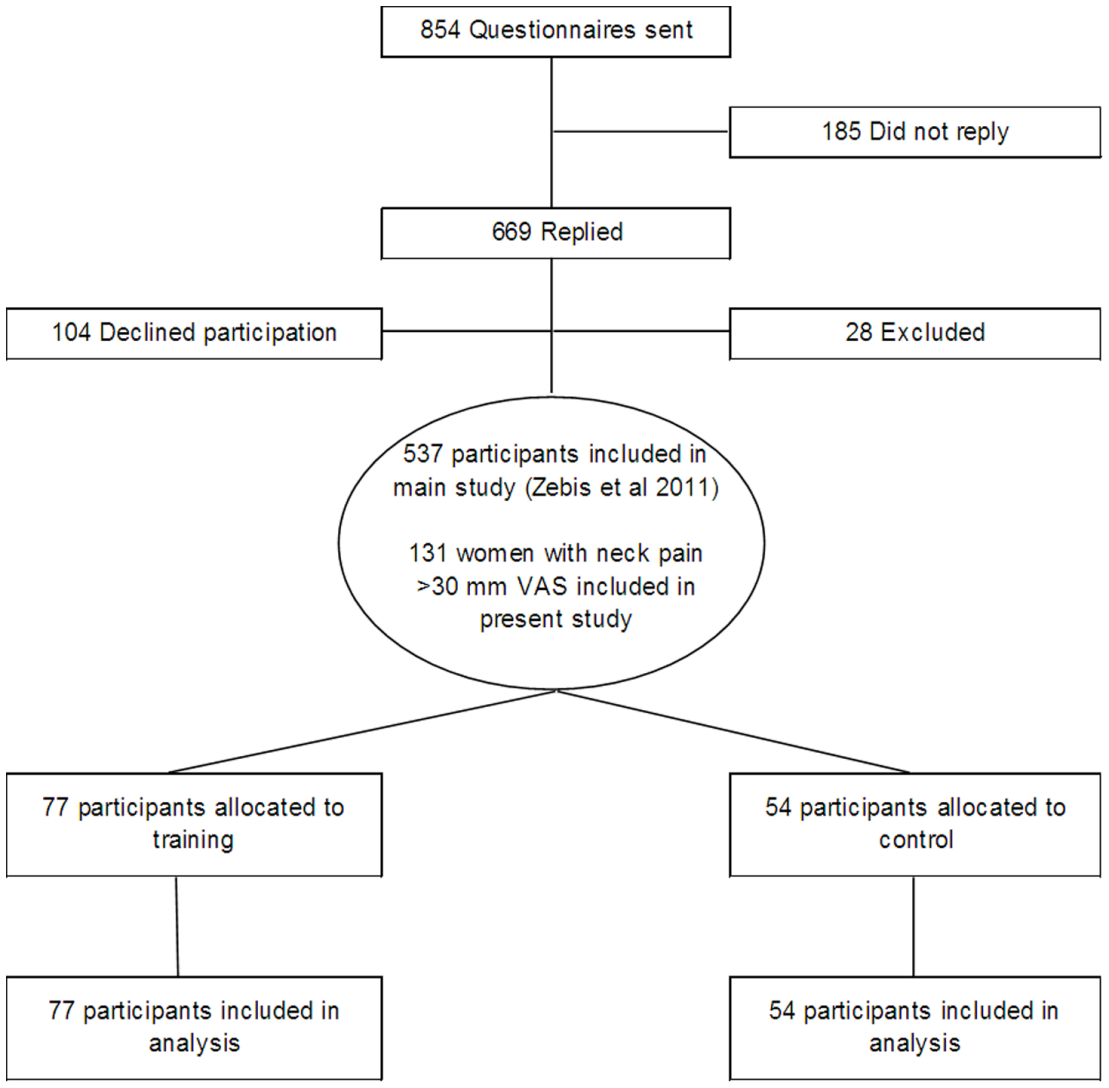
Flow of participants through the study.

The present paper is a subgroup analysis nested in the above mentioned RCT study that enrolled 82 males and 455 females (Figure 1). The present analyses included all females with a pain rating in the neck of at least 30 mm VAS at baseline (N = 131). Baseline characteristics of the 54 participants in the control group and the 77 participants in the training group are shown in Table 1.

**Table 1:** Baseline characteristics of the participants in the control group and training group, given as means (SD).

	Control (N = 54)	Training (N = 77)
Height (cm)	169 (6)	168 (6)
Weight (kg)	73 (15)	69 (13)
BMI	25 (5)	24 (4)
Age	43 (10)	42 (11)
Weekly working hours	34 (10)	35 (10)
Neck pain intensity (0–100 mm VAS)	55 (22)	56 (24)

### Intervention

The intervention took place over a 20-week period - from January to June. The participants in the training group were allowed to use a total of one hour a week during work hours for the specific training program. The training group performed high-intensity specific strength training for the neck and shoulder muscles with 4 different dumbbell exercises (front raise, lateral raise, reverse flies and shrugs) and 1 exercise for the wrist extensor muscles [25]. The training regime consisted of three sessions per week, each lasting approximately 20 minutes. During the intervention period the training load was progressively increased according to the principle of progressive overload [20] and both linear (week 1–12) and undulating periodization (week 13–20) strategies were used throughout the training program [26], [27]. After two introductory training sessions - where the exercises were explained and shown to the participants and corrections to both form and load were given - relative loadings were progressively increased from 15 repetitions maximum (RM; ∼70% of maximal intensity) at the beginning of the training period to 8–12 RM (∼75–85% of maximal intensity) during the later phase. The strengthening exercises were performed using consecutive concentric and eccentric muscle contractions with slow to moderate lifting velocity previously shown to reduce neck and shoulder pain in office workers with trapezius myalgia [16]. The participants were offered supervision in 50% of the training sessions, corresponding to every second training session.

The participants in the control group received advice to stay physically active. To secure that the control group received the same amount of attention as the training group, the participants were consulted once a week by a supervisor during the 20-week period. After the 20 week intervention period, the control group was offered an equivalent 20 week training period – i.e. 1 hour a week during work hours.

### Pain intensity

The participants in both the training and the control group were given a personal logbook and requested to register “worst pain within the last week” on a 100 mm VAS scale once a week during the 20-week intervention period. They were asked to fill out the registration at the same time on the same day each week.

### Training load and motivation

The participants in the training group registered the training load (Kg) and training motivation (rated on a 0–4 scale) for each training session in a specialized part of their logbook during the 20 week intervention period.

### Adherence

Weekly adherence to training was defined as participating at least once a week during the 20 week intervention [12]. Adherence for each participant was defined by the weekly registrations of training sessions in the personal logbook during the 20-week period.

### Statistical analyses

We performed all analyses in accordance with the intention-to-treat principle, and used analysis of variance (Mixed procedure of SAS) to determine between-group differences in neck pain. *Group* (training and control) and *time* (weeks 1–20) and *group by time* interaction was entered in the model. Missing values were imputed by last observation carried forward and first observation carried backward.

We used the SAS statistical software for the analyses (SAS institute, Cary, NC, version 9.2), and accepted an alpha level of 5% as statistically significant. We report baseline results as means (SD) and changes from baseline to follow-up as means (95% confidence intervals (CI)) unless otherwise stated.

We performed a power analysis showing that to obtain 80% power at 5% type I error risk for showing a minimal relevant difference of 10 mm VAS would require 36 participants in each group using an unpaired design.

## Results

Prior to the intervention, the subjects in the 2 groups did not differ with regard to anthropometric measurements, age, working hours and level of neck pain intensity (Table 1). At baseline the average neck pain intensity on the VAS scale was 55 mm (SD 23).

**Figure 2:**
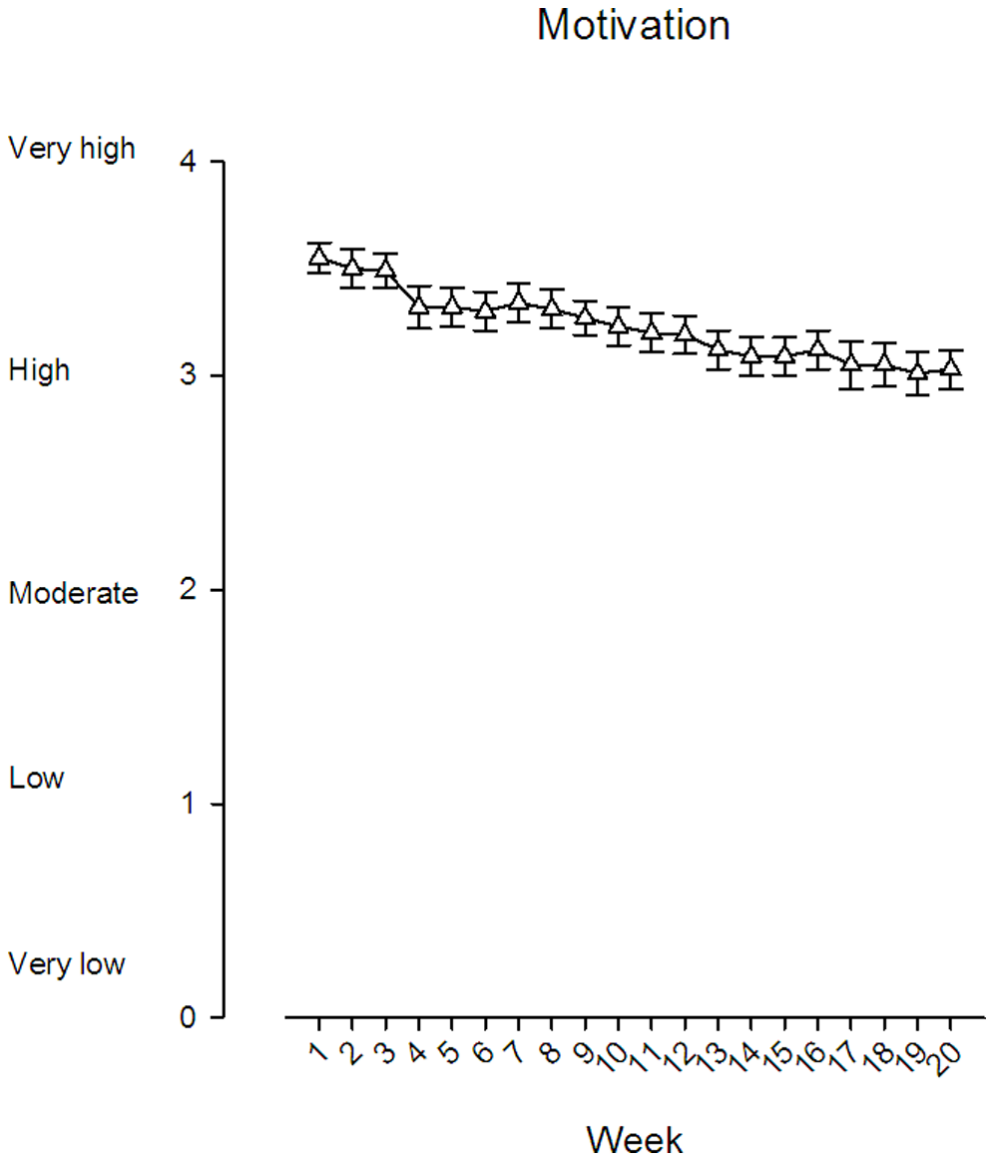
Self-reported training motivation at each session during the 20 week intervention period. Values are least square means and SE.

The training load in the training group increased with time, and was doubled by the end of the 20 weeks of training. Training motivation was rated as high all through the intervention period (first week 3.6±0.6, last week 3.2±0.8; time-wise change illustrated in Figure 2).

**Figure 3:**
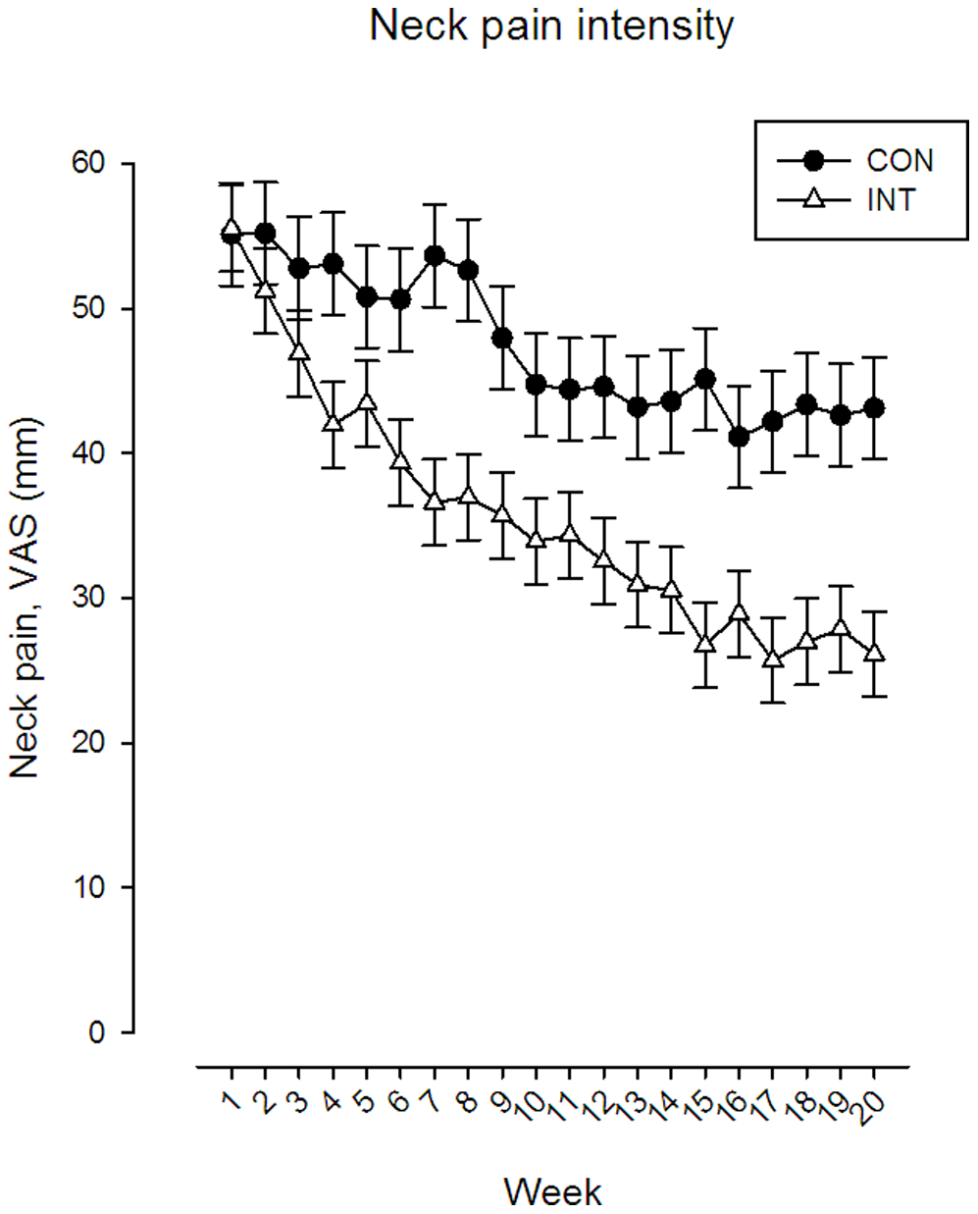
Change in neck pain during the 20 week period for the intervention and control group, respectively. The time-wise change in pain showed three phases. Values are least square means and SE.

Figure 3 shows the time-wise change in neck pain in each group throughout the 20 week period. The *group by time* interaction for neck pain was significant (F-value 2.61, P<0.001, DF = 19). From baseline to follow-up pain intensity decreased significantly more in the training group than the control group, with a between-group difference of 17 mm VAS (95% CI 8 to 26 mm VAS). A significant between-group difference in pain of 11 mm (95% CI 2 to 20 mm VAS) was seen after 4 weeks.

The time-wise change in pain showed three phases; Phase I: A rapid decrease in pain in the training group compared with the control group during the initial 7 weeks, corresponding to a between-group difference in pain of 17 mm (95% CI 8 to 26 mm VAS) (P<0.001) - and a decrease in neck pain intensity from 55 mm (SE 3) at baseline to 37 mm (SE 3) at week 7 in the training group. Phase II: A slower decrease in pain during week 8–15 with a between-group difference in pain of 18 mm (95% CI 9 to 27 mm VAS) (P<0.001), corresponding to an average neck pain intensity of 27 mm (SE 3) in the training group at week 15. Phase III: A plateau from week 16 to the end of intervention where no further decrease was observed in the training group compared with the control group.

**Figure 4:**
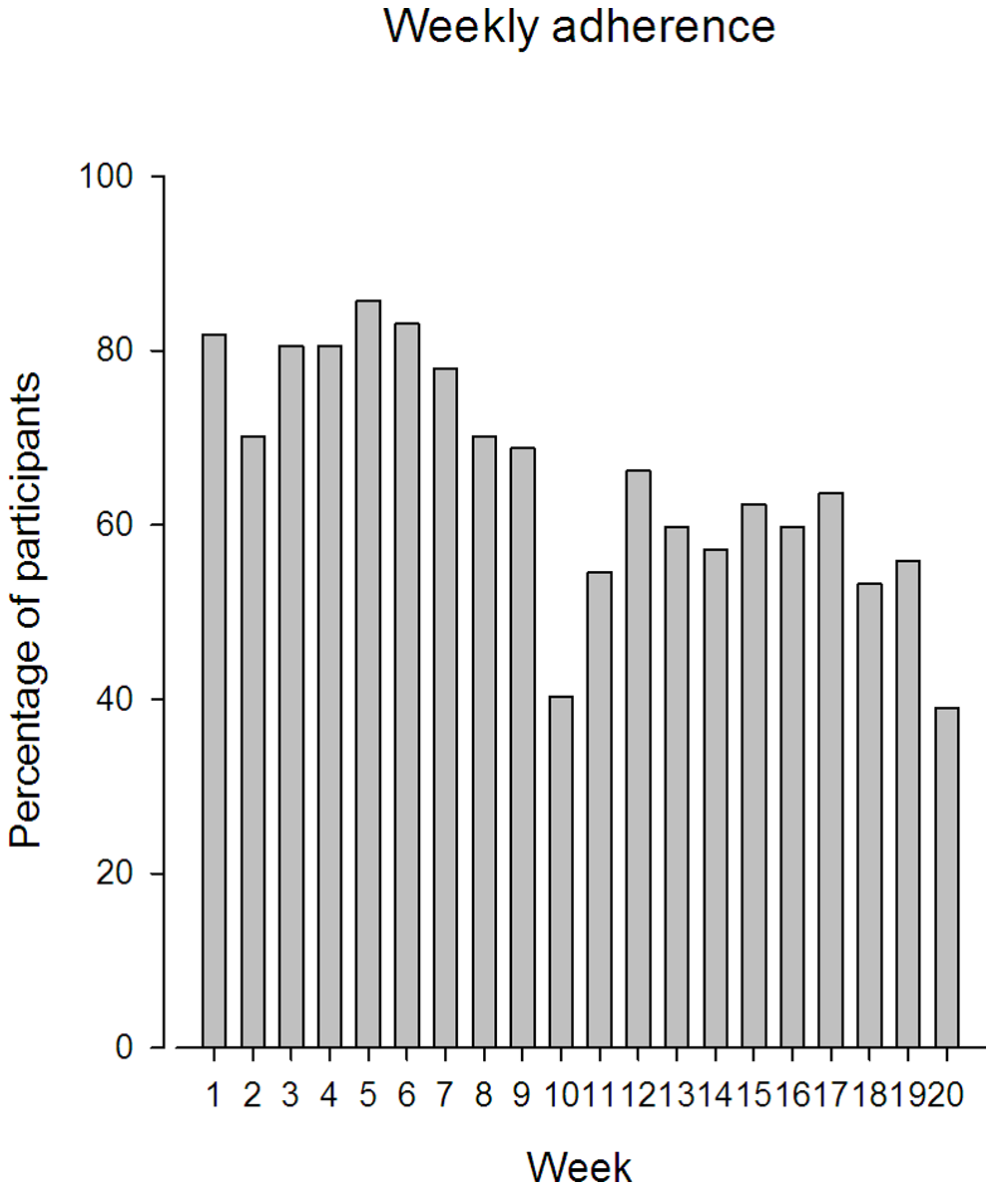
Weekly adherence to training during the 20 week intervention period in the training group.

Figure 4 shows that adherence to training followed a two-phase pattern. Weekly participation rate was between 70–86% during the initial 7 weeks, dropping towards 55–63% during the latter half of the training period.

## Discussion

Our study showed that specific strength training effectively reduced neck pain among female industrial workers. Relevant changes were observed after only 4 weeks of strength training indicating that this amount of time is necessary to achieve a minimal clinical relevant pain reduction [23], [28]. Further decrease in pain intensity was seen until week 16 with the steepest slope on the neck pain/time curve – i.e. fastest decrease in pain intensity - during the initial 7 weeks of training Adherence to training was highest during the initial 7 weeks of training after which adherence to training was decreasing – especially after week 10 which coincided with a national vacation week. This implied that following holidays, adherence to training was challenged and special efforts may be necessary to keep adherence high. Although adherence to exercise decreased during the latter phase, training motivation remained high all through the intervention period.

The strength training protocol used in the present study has previously been shown to rapidly and effectively reduce neck pain and improve muscle function in office workers with trapezius myalgia [16], [21]. Thus, Andersen et al. showed that 10 weeks of high intensity strength training induced a linearly time-wise decrease in pain intensity by 79% from baseline in women with chronic neck pain [16]. Consequently, the observed significant and clinically relevant neck pain reduction in the present study was expected. In our study, the initial 10 weeks of training decreased pain intensity by 36% from baseline, and by 49% at the end of intervention. The less significant pain reduction seen after 10 weeks of training in the present study may partly be explained by a somewhat more heterogeneous pain group than in the study by Andersen et al. (2009) where only women with diagnosed trapezius myalgia were included and all training sessions was closely supervised.

Contrary to the intensive short term intervention by Andersen et al. [16], a plateau in neck pain reduction was reached during the present 20 week intervention period. Thus, our study showed a time period – i.e. 15 week - after which no further reductions in pain were seen. However, weekly adherence to training was achieved by only 60% of the participants during the last 5 weeks which may explain the decrease in efficacy during the last phase of the intervention. Although the observed plateau may be explained by low adherence, the finding suggest that phase I (week 1–7) and II (week 8–15) result in minimal (17 mm) to moderate (27 mm) clinical significant relevant neck pain reductions, respectively, with improvements maintained throughout the last phase of the training period (week 16–20). Consequently, it could be stated that the present “exercise dose” taken in a 15 week period will be sufficient to reach an optimal neck pain reduction - presupposing that weekly adherence to training is achieved.

In the control group a decrease in neck pain intensity was observed in the initial 10 weeks. This observation may be explained by the fact that chronic neck pain symptoms are known to display seasonal variation, worsening in the autumn and decreasing in the spring [29]. Since the present study took place from January to June, a general decrease in neck pain symptoms was expected.

In the present study we did not investigate the underlying mechanisms of pain reduction. However, we have previously tested the effect of similar types of exercise in a small group of women with chronic neck muscle pain (trapezius myalgia). In that study, the women with chronic neck muscle pain had a marked pain-inhibition of muscle function as measured by isokinetic dynamometry and electromyography [30], [31]. After the 10 week strength training intervention pain was reduced [32] and muscle strength increased as expected in response to strength training [33], but more importantly the ability to rapidly develop muscle force almost doubled [34]. The mechanisms responsible may not only be physiological, but could partly be explained by distinct psychological factors. That is fear avoidance of movement as often observed in pain patients, leading to slow and stiff movements, was abolished after the strength training period. Thus, strength training reduces pain, and also restores muscle function and the ability to use the body in a more natural way. From a physiological point of view the high intensity strength training exercises can increase blood flow to the painful muscles and enhance protein turnover of muscle tissue, which may aid recovery.

## Conclusions

In conclusion, specific resistance training effectively reduced neck pain among female industrial technicians with frequent neck/shoulder pain symptoms. During a 20 week intervention period, the time-wise change in pain followed a three-phase pattern with a rapid effect during the initial weeks followed by a slower effect during the latter phase, and finally a plateau during the last 5 weeks. Decreased participation rate may explain the decrease in efficacy during the latter phase of the intervention.
